# Internalization of External Benefits Brought by Hydropower Development

**DOI:** 10.3390/ijerph17010338

**Published:** 2020-01-03

**Authors:** Huiyan Wang, Yong Li, Jia Li, Mengyuan Yu

**Affiliations:** 1 State Key Laboratory of Hydraulics and Mountain River Engineering, Sichuan University, Chengdu 610065, China; why@mail.xhu.edu.cn (H.W.);; 2 Key Laboratory of Fluid and Power Machinery, Ministry of Education, Xihua University, Chengdu 610039, China; 3 College of Environment, Hehai University, Nanjing 210098, China

**Keywords:** hydropower development, external benefit, internalization, compensation standard

## Abstract

Hydropower development brings a very large number of external benefits which are enjoyed by the beneficiaries for free. These external benefits are defined and the beneficiaries are identified. Models to measure the external benefits are established to reflect their dynamic changes at different periods. To improve the benefit sharing mechanism, a model to internalize these external benefits is established to further compensate those adversely affected. The Z hydropower project in China is taken as the example to calculate its external benefits and their internalization. The external benefits enjoyed by beneficiaries in the surrounding and downstream areas gradually increase from 18 million US dollars in 2006 to 114 million US dollars in 2065, and their compensation standards increase from 4 million US dollars in 2006 to 97 million US dollars in 2065. The external benefits enjoyed by beneficiaries in the power receiving areas increase from 125 million US dollars in 2015 to the maximum of 133 million in 2026, and their compensation standards increase from 38 million US dollars in 2015 to the maximum of 133 million US dollars in 2033. Sharing of external benefits can improve the benefit-sharing mechanism, and properly redistribute the external benefits of hydropower development.

## 1. Introduction

The installed capacity of hydropower in China has exceeded 350 GW and the annual hydropower generation has exceeded 120 TWh, and both of which are ranking the first in the world [1]. However, with the rising difficulty of resettlement, the increasing pressure on ecological protection, and the declining economic efficiency of hydropower development, the competitive advantage of hydropower in the energy market is getting smaller and smaller. Furthermore, as a clean and renewable energy, the preferential access of hydropower to the grid has not been guaranteed by national laws and systems, and the abandonment of hydropower is a serious problem in China [2]. How to deal with the plight faced by hydropower development, how to further mitigate the social and ecological problems caused by hydropower development, how to guarantee the healthy, orderly, and sustainable development of hydropower, and how to realize the environment friendly, benefit sharing goal of hydropower development, are the problems urgently need to be solved in the new era.

Scholars around the world have tried to solve these problems. Costanza (1997) estimated the economic value of 17 ecosystem services for biomes [3], and based on these values, other scholars used the payment for ecosystem services (PES) [4,5] or the ecological compensation [6,7,8] to internalize the external impacts on the environment by hydropower development. Fan Qixiang (2010) established a benefit-sharing model based on the values of resources and the theory of land rent to solve the resettlement problems [9]. Shrestha (2016) summarized the benefit-sharing mechanisms in Nepal into five types and provided specific recommendations for each type of mechanism [10]. Branche E. (2017) constructed a “sharing” concept to maximize the benefits of multi-functional hydropower projects such as power generation, water supply, entertainment, ecological services, and economic growth through vision sharing, resource sharing, responsibility sharing, rights and risks sharing, as well as cost and benefit sharing among stakeholders [11]. Rayamajhee (2018) designed an endogenous externality mitigation fund based on local negotiations, which is directly used to compensate individuals affected by specific environmental externalities, to replace the one-off tax policies and measures to include external costs in energy prices [12].

However, these studies are mainly focused on the negative impacts of hydropower development, and only emphasized the benefit sharing by hydropower development enterprises. While the studies on positive impacts are a few, and even fewer on the internalization of positive impacts, such as optimizing the energy structure, improving the atmospheric environment and stimulating the economic development. These positive impacts are enjoyed by beneficiaries free of charge. Facing with the plight of hydropower development, it is impossible for hydropower development enterprises to deal with the above problems alone, so the beneficiaries of hydropower development shall also share their external benefits. In this paper, the theory of externality is introduced into the evaluation of hydropower development, the external benefits brought by hydropower development are defined and measured, the external beneficiaries are identified and their compensation standards are calculated. The internalization model established can help to improve the traditional benefit sharing mechanism by allowing the beneficiaries of hydropower development besides the enterprise developing the project to share their external benefits. In this way, the benefits of hydropower development including the internal and external benefits can be properly distributed, the pressure on hydropower development enterprise can be relieved, and those poorly affected can be further compensated, which will lay a solid foundation for the coordinated and sustainable development of hydropower development.

## 2. Materials and Methods

### 2.1. Theory of Externality and Internalization

Externality is the cost or benefit that affects a party who did not choose to incur that cost or benefit [13]. Externality hides the true cost or benefit of a product and leads to the tragedy of the commons. To internalize the externalities, Pigou believed that externalities are caused by market failure, and the optimal allocation of social resources can only be realized through government intervention [14]. Coase pointed out that the root of externalities lies in the improper or unclear definition of property rights, and believed that externalities can be resolved through voluntary negotiated market transactions after the definition of property rights [15]. Greenwald and Stiglitz pointed out that, in the real world, due to the lack of insufficient information among market participants, the function of the market is not perfect, which will harm people’s interests. Therefore, the government’s intervention in the market is the key to ensure the normal operation of the market [16]. In a word, neither the government nor the market alone can solve the problem of externalities, so we must take full advantage of both sides and ensure the normal operation of the market through clever intervention by the government. The market mechanism is used to measure the size of the external benefits and to calculate the compensation standard after enjoying the external benefits; the government mechanism is used to urge the external beneficiaries to share their external benefits to internalize the externalities of hydropower development.

### 2.2. Externality Measurement Model

The external benefits of hydropower development refer to the benefits brought by the hydropower development enterprises during the development of hydropower, but is enjoyed by other entities rather than the hydropower development enterprises. The external benefits include stimulating the development of regional economy, upgrading the shipping performance, improving the grid performance, regulating the local climate, improving the atmospheric environment, increasing the environmental capacity of water, etc. The beneficiaries of these external benefits are governments, enterprises, and individuals in the area surrounding the project, the area downstream of the project and the area receiving the power supply. These external benefits can be calculated by the following equation:

(1) EX=RE+SP+GP+LC+AE+WE.

where *EX* represents the external benefits brought by the development of hydropower project; *RE* represents the external benefit in stimulating the development of regional economy; *SP* represents the external benefit in upgrading the shipping performance; *GP* represents the external benefit in improving the grid performance; *LC* represents the external benefit in regulating the local climate; *AE* represents the external benefit in improving the atmospheric environment; and *WE* represents the external benefit in improving the environmental capacity of water.

#### 2.2.1. External Benefit in Stimulating the Development of Regional Economy

This refers to the value of stimulating the economic development of the surrounding areas and the areas receiving electricity during the construction and operation period of a hydropower project. Due to the abundant power supply in China, the stimulation of the economy in the areas receiving electricity is neglected. The economic development of the surrounding areas is reflected in the growth of regional economy, the provision of more employment opportunities, the increasing of incomes, etc. The total output of regional economic growth mainly consists of cost, profit and tax. While the former two have been internalized through marketization, the tax is the embodiment of the external benefit. So, the external benefit in stimulating the development of regional economy can be calculated by the following equation:

(2) RE=Tax×FIRR+N×W.

where *Tax* represents the tax revenue brought by hydropower development to the local government during the construction and operation periods, million US dollars/year; *FIRR* represents the financial internal rate of return; *W* represents the average salary of the job provided during the construction period; *N* represents the number of employment opportunities provided during the construction period, which can be calculated by using the coefficient of employment elasticity [17].

#### 2.2.2. External Benefit in Upgrading the Shipping Performance

This refers to the value of providing or improving the navigation conditions. It includes the benefits of transportation cost saving, transportation efficiency and shipping quality improving when compared with that before the project development, as follows:

(3) $$SP=H_{1}+H_{2}+H_{3}$$

where $H_{1}$, $H_{2}$, and $H_{3}$ represent the benefits of transportation cost saving, transportation efficiency and shipping quality improving, separately, in million US dollars/year.

(4) $$H_{1}=L_{y}Q_{hn}\left(C_{hw}-C_{hy}\right)+\left(Q_{ht}+\frac{1}{2}Q_{hi}\right)\left(C_{ho}L_{o}-C_{hy}L_{y}\right)$$

where $C_{hw}$, $C_{ho}$ and $C_{hy}$ represent the unit freight without the project, of the original relevant route and with the project, separately, in US dollars/(t·km), US dollars/(capita·km); $L_{y}$. and $L_{o}$. represent the transportation distances with the project and of the original relevant route, in km; $Q_{hn}$, $Q_{ht}$, and $Q_{hi}$ represent the normal traffic, transfer traffic and induced traffic, in million t/year, million capita/year.

(5) $$H_{2}=\frac{1}{2}\left(T_{hnp}Q_{hnp}+T_{htp}Q_{htp}\right)b_{p}+P_{cargo}Q_{hc}T_{st}+C_{dm}T_{sd}q_{s}$$

where $T_{hnp}$ and $T_{htp}$ represent the time saved for passengers in normal and transfer passenger transports, in h/capita; $Q_{hnp}$ and $Q_{htp}$ represent the number of production personnel in normal and transfer passenger transports, in million capita/year; $b_{p}$. represents the unit value of passenger’s time, in US dollars/h; $P_{cargo}$ represents the value of cargos, in US dollars/t; $Q_{hc}$ represents the cargo traffic volume, in million t/year; $T_{st}$ represents the transportation time saved by the project, in h; $C_{dm}$ represents the daily maintenance cost of the ship, in million US dollars/(ship· day); $T_{sd}$ represents the detention time saved for ships per year, in days; and $q_{s}$ represents the number of ships.

(6) $$H_{3}=\gamma Q_{hc}P_{cargo}+P_{ha}Q_{hc}\Delta J+H_{31}$$

where γ and ΔJ represent the reduction rate of shipping cargo loss and shipping accidents, separately; $P_{ha}$ represents the average loss in maritime accidents, in million US dollars/time; $H_{31}$ represents the cost saved from navigating in difficult and torrent flows, in million US dollars/year.

#### 2.2.3. External Benefit in Improving the Grid Performance

This refers to the part of value that has not been compensated for the loss of hydropower generation caused by the provision of rotating reserve and emergency reserve for the grid. People can benefit from the grid stability. This benefit can be calculated by the capacity cost, annual operating cost, and incremental cost of thermal power units that are saved when they are replaced by hydrogenating units to provide rotating reserve and emergency reserve, as follows:

(7) $$GP=G_{1}+G_{2}+G_{3}-E_{w}$$

where $G_{1}$, $G_{2}$, and $G_{3}$ represent the capacity cost, the annual operating cost and the incremental cost of coal when using thermal power units for rotating and emergency reserve, in million US dollars/year; $E_{w}$ represents the annual operation cost of using hydrogenating units, in million US dollars/year.

(8) $$G_{1}=\Delta CK_{H}\left[1+\left(P/F,i_{1},n_{1}\right)\right]\left(A/P,i_{1},n_{2}\right)$$

where ΔC represents the spare capacity of the thermal power unit corresponding to the hydrogenating unit, in kW; $K_{H}$ represents the per kW investment of thermal power, in million US dollars/kW; $P/F,i_{1},n_{1}$ represents the present value factor of compound interest with the discount rate as $i_{1}$ and the number of periods as *n*_1_; $A/P,i_{1},n_{2}$ represents the inverse of the annuity present value coefficient with the discount rate as $i_{1}$ and the number of periods as *n*_2_; $i_{1}$ represents the social rate of discount; $n_{1}$ represents the life span of the thermal power station, in years; $n_{2}$ represents the life span of the hydropower station, in years.

(9) $$G_{2}=R_{v}\frac{\Delta C}{\alpha }K_{H}-R_{c}\left(1-\alpha \right)\frac{\Delta C}{\alpha }K_{H}$$

where $R_{v}$ represents the rate of variable output operation; 1−α represents the minimum technical output factor of thermal power unit; $R_{c}$ represents the rate of stable operation.

(10) $$G_{3}=\left(1-\alpha \right)\frac{\Delta C}{\alpha }\left(365-T_{o}\right)24\left(b_{y}-b_{e}\right)P_{coal}$$

where $T_{o}$ represents the annual maintenance time of the power station, in days; $b_{y}$ represents the coal consumption at the non-rated power, in g/kW·h; $b_{e}$ represents the coal consumption at the rated power, in g/kW·h; $P_{coal}$ represents the price of standard coal, in US dollars/t.

(11) $$E_{w}=A_{w}r_{w}f_{w}$$

where $A_{w}$ represents the static investment of the hydropower station, in millions of US dollar; $r_{w}$ represents the annual operation rate of the hydropower station; $f_{w}$ represents the ratio of spare capacity.

#### 2.2.4. External Benefit in Regulating the Local Climate

This refers to the value of promoting the growth and reproduction of plants and animals by local climate regulating from the large body of water. People can benefit from the products of climate regulation. This benefit can be calculated by the ecological service value of water ecosystem per unit area [18]:

(12) $$LC=\Delta A_{water}\times P_{water}\times \frac{1}{1+e^{m-nx}}$$

where $\cdot A_{water}$ represents the difference of water surface areas before and after reservoir formation, in hm^2^; $P_{water}$ represents the value of ecological services per unit area of aquatic ecosystem, in US dollars/hm^2^; m, n represent constants related to the plant growth rate of typical vegetation around the reservoir in the year *x* after impounding.

#### 2.2.5. External Benefit in Improving the Atmospheric Environment

This refers to the value of reducing the emission of carbon dioxide and other exhausts through replacing thermal power with hydropower. People will benefit from the good air quality. This benefit can be reflected by the transaction cost or treatment cost of carbon dioxide and sulfur dioxide emitted if using thermal power instead of hydropower power and minus the carbon dioxide emitted from the reservoir, as follows:

(13) $$AE=R_{H}\times C_{coal}\times \left(Q_{CO2}\times P_{CO2}+Q_{SO2}\times P_{SO2}\right)-\frac{12}{m}\times V\times 10\times \overset{m}{\underset{i=1}{\sum }}\left[6.11CH_{4}\left(m\right)+22.5\right]$$

where $R_{H}$ represents the annual output of the hydropower project, in million kW·h;$\;C_{coal}$ represents the average power consumption of coal-fired power generating units, in g/kW·h; $Q_{CO2}$ and $Q_{SO2}$ represent the amount of CO_2_ and SO_2_ emission per ton of standard coal combustion [19], in tCO_2_/tce, rSO_2_/tce; $P_{CO2}$ represents the transaction cost per ton of CO_2_, in US dollars/t, determined by the local carbon trading market value; $P_{SO2}$ represents the treatment cost per ton of SO_2_ [20], in US dollars/t; *m* represents the number of months calculated at the start of impoundment; *V* represents the volume of reservoir, in m^3^; *CH*_4_(*m*) represents the content of dissolved methane in water, in mg/L, which is calculated by using the Delmas model [21] as $CH_{4}\left(m\right)=\left[10.5+3.5\cos(2\pi /12\right)m]exp^{-0.015m}$.

#### 2.2.6. External Benefit in Increasing the Environmental Capacity of Water

This refers to the value of increasing the risk prevention and control ability of water bodies through changing the environmental carrying capacity. People will benefit from the good water quality. This benefit can be reflected by the changes of the environmental capacity of water, as follows:

(14) $$V_{szo}=\overset{n}{\underset{i}{\sum }}\left(JA_{i}-JB_{i}\right)\times X_{i}\times 365$$

where *i* represents the pollution control factors, such as ammonia nitrogen, TP, TN, COD, etc.; *JB_i_*, *JA_i_* represent the environmental capacities of various pollution control factors before and after reservoir formation, in t/d; *X_i_* represents the transaction cost or disposal cost per unit discharge of each pollution control factor, in US dollars/t.

### 2.3. Internalization of External Benefit

According to the theory of externality, the benefit sharing mechanism goes beyond the obligatory requirements of the current resettlement and compensation measures, environmental protection measures, soil and water conservation measures, etc. The external beneficiaries shall also share their external benefits besides the development enterprises sharing their project benefits to fully realize the benefit sharing of hydropower development and restore the function of the natural ecosystem.

#### 2.3.1. Internalization Model of External Benefits

By combining the external benefits and the adjustment coefficient, a dynamic compensation standard model is constructed for the external beneficiaries of hydropower development to share their benefits and compensate those adversely affected by hydropower development:

(15) $$CS_{x}=\left[\overset{n}{\underset{j=1}{\sum }}EP_{xj}\times I_{xj},\overset{n}{\underset{j=1}{\sum }}EP_{xj}\right]$$

where *CS_x_* represents the compensation standard which shall be provided by the beneficiaries after enjoying the external benefits of hydropower development in year *x*, in millions of US dollars/year; *EP_xj_* represents the external benefit brought by the hydropower development for the beneficiaries in benefiting area *j* in year *x*, in millions of US dollars/year; *I_xj_* represents the adjustment coefficient of the compensation standard provided by the benefiting area *j* in year *x*.

This compensation model is a standard compensation interval. The maximum value of the interval is *EP*, which is the external benefit enjoyed by beneficiaries in the process of hydropower development. It mainly includes the external benefit of stimulating the regional economy during the construction period, and the other five external benefits during the operation period. If using *EP* as the compensation standard, the beneficiaries cannot keep any of the external benefits, which clearly is an extreme value, and the actual compensation value is also affected by many other factors. The minimum value of this interval is the product of the maximum value *EP* and the adjustment coefficient *I*. The adjustment coefficient is affected by the development status, the ability to pay, the willingness to pay, and many other factors related to the beneficiaries. It is used to determine the minimum amount of compensation that external beneficiaries shall pay in order to achieve a relatively fair distribution of benefits at a specific stage of development. Compensating within this range not only can provide financial support to eliminate the negative externalities of the hydropower development, and guarantee the external beneficiaries to share their external benefits within the scope of their ability to pay, but can also stimulate the enthusiasm of hydropower development enterprises in helping those adversely affected by hydropower development.

#### 2.3.2. Determination of the Internalization Adjustment Coefficient

With the development of economy, the progress of society, the improvement of living standards and the increasing awareness of environmental protection, people’s willingness and ability to pay for the external benefits they enjoyed will continue to increase. Therefore, the internalization adjustment coefficient can be predicted by the social development, the living standard of the beneficiaries and the degree of environmental protection awareness. Engel’s coefficient is the proportion of total food expenditure in the total personal consumption expenditure. It is an important international index to measure the living standard of residents. It generally decreases with the increase of household income and living standard [22]. Although there are many other indicators to measure the living standards of residents, Engel’s coefficient can largely reflect the level of living standards. In addition, Engel’s coefficient can be easily found in relevant statistical websites or yearbooks, which has a good availability, so using Engel’s coefficient to determine the adjustment coefficient has a good reproducibility. Therefore, this paper uses Engel’s coefficient to determine the internalization adjustment coefficient, as follows:

(16) $$I_{x}=\frac{1}{1+e^{-\left(\frac{1}{En_{x}}-3\right)}}$$

where *e* represents the napierian base; *En* represents the Engel’s coefficient in year *x*; 3 is the accommodation factor.

This adjustment coefficient is the compensation standard that external beneficiaries shall provide after enjoying the external benefits of hydropower development. After compensation, the economic development of the beneficiaries will not be affected, meanwhile more compensation can be provided for those adversely affected so as to ensure their development and to restore the natural ecological environment. When the Engel’s coefficient in the benefiting area approaches 0, the adjustment coefficient tends to be 1. That is, when the social development level is extremely high and people are extremely rich, people will pay more attention to environment protection. In order to make the overall environment better, the beneficiaries will take the initiative to use all the external benefits to compensate those adversely affected. When the Engel’s coefficient in the benefiting area is approaching 1, the adjustment coefficient tends to be 0. That is, when the social development level is extremely low and people are very poor, people will pay more attention to economic development and may neglect the protection of environment, and they are not willing to share the external benefits they enjoyed. When the Engel’s coefficient is between 0 and 1, people’s willingness to pay is positively correlated with the social development level and people’s degree of affluence, that is, it is negatively correlated with the Engel’s coefficient. However, the relationship between them is not linear, but after a certain extent, the influence effect is not obvious, in line with the changing pattern of s-shaped curve. By showing the amount of external benefits people enjoyed and the amount they need to share to internalize the external benefits, this model will urge external beneficiaries to share their external benefits, which will relieve the pressure on the hydropower development enterprise, and further compensate those poorly affected by the project development.

## 3. Results

The Z hydropower development project on JS River in Sichuan Province and Yunnan Province is chosen as the case to evaluate its external benefits and their internalization. The data used are collected from the Feasibility Study Report, the Environment Impact Assessment Report of Z Hydropower Station, China statistical information network, and websites of relevant statistical bureaus and governments. 

### 3.1. External Benefits Brought by the Z Hydropower Development

**Figure 1:**
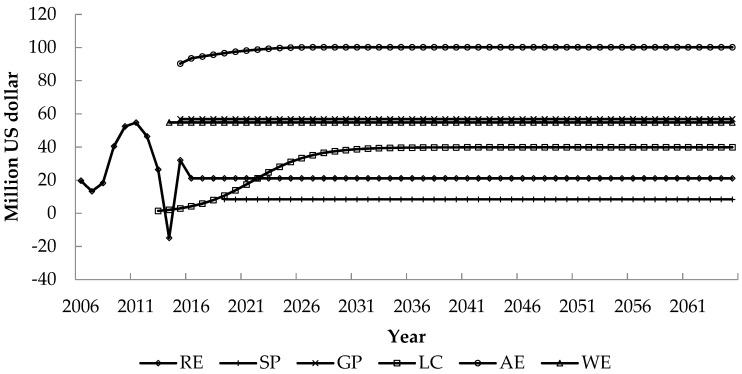
External benefits brought by the Z hydropower development.

The development of the Z hydropower project brought vast external benefits, as shown in Figure 1. The external benefit of shipping performance upgrading is the smallest, while the external benefit of atmospheric environment improvement is the largest.

The benefit of regional economy stimulation will manifest at the beginning of the project construction in 2006, it changes with the project investment and the working intensity during the construction period, and even drops to a negative benefit in 2014 due to the reduction of investment during the completion period, but remains constant at 21 million US dollars after the normal operation of the project. The benefit of shipping performance upgrading is 8 million US dollars/year on average, which will manifest after the readiness of the 24 h uninterrupted navigation capacity in 2019. The benefit of grid performance improvement is 57 million US dollars/year on average, which will manifest after the normal operation of the hydropower project in 2015. The benefit of local climate regulation manifests gradually in accordance with the biological growth pattern, and reaches to a new balance of 40 million US dollars in 2039. The benefit of atmospheric environment improvement manifests after the supply of clean power to the power receiving area in 2015. With the reducing of carbon dioxide emission from the reservoir and the reduction of carbon dioxide and sulfur dioxide emission due to the replacement of thermal power plants, this external benefit gradually increases from 90 million US dollars in 2015 to 701 million US dollars in 2026, and then tends to be constant. The benefit of water environmental capacity improvement is 55 million US dollars/year on average, which will manifest after impoundment to the normal water level in 2014. 

### 3.2. Internalization of External Benefits Brought by the Z Hydropower Development

The beneficiaries of positive externalities of the Z hydropower development are mainly distributed in the surrounding areas, downstream areas of the dam and the power receiving areas. The surrounding areas of the Z hydropower project are mainly A county of C city in Yunnan Province and B county of D city in Sichuan Province. The downstream area is mainly D city. Due to the overlap between the surrounding area and the downstream area and the similar level of economic development, these two areas are combined to consider their compensation standard. The compensation period is from the beginning of construction to the end of the externality impact calculation, that is, from 2006 to 2065. The power receiving area is mainly E City, and the compensation period is from the normal operation of the project to the end of the calculation period, that is, from 2015 to 2065.

#### 3.2.1. Compensation Standards of Beneficiaries in Surrounding Areas and Downstream Areas

Z hydropower development has brought huge external benefits to the surrounding areas (A county and B county) and the downstream areas (D City), mainly including the external benefits of regional economy stimulation, shipping performance upgrading, local climate regulation, and water environmental capacity improvement.

The main beneficiaries of the Z hydropower development in C city is A county, so the data in A county is used to predict the adjustment coefficient of C city. Although the main beneficiaries of the Z hydropower development in D city is B county, the Engel’s coefficient of B county collected over the years does not have statistical pattern, so the data of D city is used. According to the statistics yearbooks of A county and D city, as well as the statistical bulletin of national economic and social development, the Engel’s coefficients of urban residents and rural residents of A county and D city are collected and calculated, as list in Table 1.

**Figure 2:**
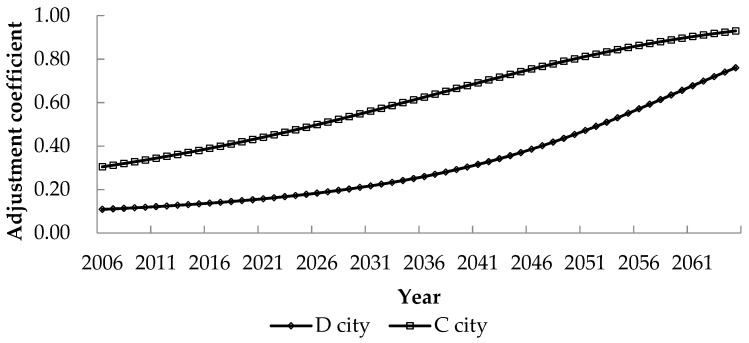
Adjustment coefficient of compensation standard for beneficiaries in C city and D city.

According to the above data, the changing trends of Engel’s coefficient of all residents in A county and D city are fitted as: $En_{C}=4E+13e^{-0.016t}$ and $En_{D}=5E+22e^{-0.026t}$, where ***R***^2^ = 0.6554 and ***R***^2^ = 0.7202, so the fitting effects are acceptable. The predicted ***En**_**C**_* and ***En**_**D**_* are substituted into Equation (16) to obtain the internal compensation standard adjustment coefficients ***I**_**C**_* of C city and ***I**_**D**_* of D city, as shown in Figure 2.

The fitting results show that the adjustment coefficient of D city is lower than that of C city, and the main reason is that the data of A county in C city and the data of D city are used for prediction. The degree of benefit in other countries besides B county in D city is lower, so their willingness to pay is low. However, the changing patterns of the adjustment coefficients for the internalization compensation standard are the same.

To combine the different adjustment coefficients of the two cities involved, the distribution proportion of water resource cost of the Z hydropower project in Sichuan and Yunnan is taken as the weighted average (50.49% in Yunnan Province and 49.51% in Sichuan Province). Thus, the adjustment coefficient of the surrounding and downstream areas $I_{CD}=I_{C}\times 50.94\%+I_{D}\times 49.51\%$. According to Equation (15), the compensation standards provided by C city and D city are as follows:

(17) $$CS_{CDx}=\left[\left({\left(RE+SP+LC+WE\right)}_{x},I_{CDx}\right),{\left(RE+SP+LC+WE\right)}_{x}\right]$$

**Figure 3:**
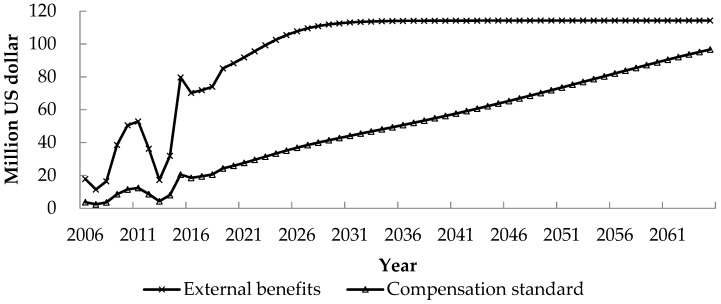
External benefits enjoyed and compensation standards provided by beneficiaries in C and D cities.

The changing trends of the external benefits enjoyed by C city and D city brought by the Z hydropower development and their compensation standards after enjoying the external benefits are shown in Figure 3.

The development of the Z hydropower project will bring several external benefits for the surrounding areas of the project and the downstream areas of the dam. Due to the fluctuation of the benefit in regional economy stimulation and the gradual manifestation of the benefit in local climate regulation, the external benefits enjoyed by C and D cities fluctuate during the construction period, but during the normal operation period, they are increasing gradually, from 18 million US dollars in 2006 to 114 million US dollars in 2065. After enjoying these external benefits, the beneficiaries in both areas shall share their external benefits and compensate those adversely affected by the project. With the awareness of the external benefits they enjoyed and the development of the society, their willingness and ability to compensate increase gradually. The adjusted compensation standard increases gradually from 4 million US dollars in 2006 to 97 million US dollars in 2065.

#### 3.2.2. Compensation Standards of Beneficiaries in Power-Receiving Areas

The Z hydropower development has brought huge external benefits to the power receiving areas, including the improvement of the grid performance and the improvement of atmospheric environment. Z hydropower station exports 80% to 90% of its electric energy production to E city via a DC line, so only the compensation standard that E city, the main power receiving area, should provide is considered.

According to the statistical yearbook of E city and the proportion of residents’ per capita consumption expenditure, the Engel’s coefficients of E residents from 2012 to 2017 are collected as listed in Table 2.

According to the above data, the changing trend of Engel’s coefficient of the residents in E city is fitted as: $En_{E}=2E+79e^{-0.091t}$, where ***R***^2^ = 0.8795, so the fitting effect is acceptable.

**Figure 4:**
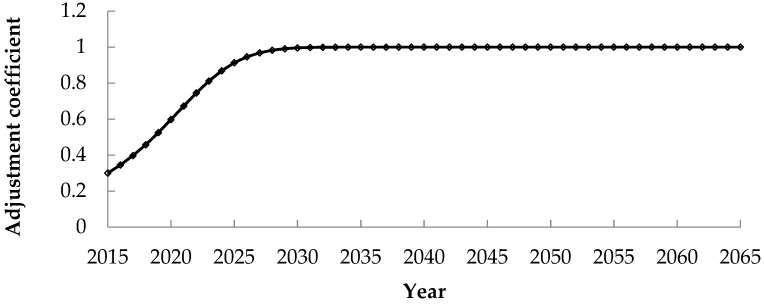
Adjustment coefficient of compensation standard for beneficiaries in E city.

The predicted ***En_E_*** is substituted into Equation (16) to obtain the internal compensation standard adjustment coefficient ***I_E_*** of E city, as shown in Figure 4.

According to the fitting results, the adjustment coefficient of E city is higher than that of C and D cities, because the beneficiaries in E city have a high level of social development and strong ability to pay. With the development of the society, beneficiaries in E city have higher and higher recognition of the external benefits brought by the Z hydropower development, and their payment capacity and willingness to pay are also growing quickly. After 2038, the adjustment coefficient tends to be 1. That is, the beneficiaries in E city have the ability and willingness to take out all the external benefits they enjoyed for the restoration and protection of the natural ecological environment in the project development area.

Only 80%–90% of the power generated by the Z hydropower project is sent to E city, so the external benefits that E city enjoys from grid performance and atmospheric environment improvement are calculated as 85% of the total external benefits. According to Equation (15), the compensation standard to be provided by E city is as follows:

(18) $$CS_{Ex}=\left[\left({\left(GP+AE\right)}_{x}\cdot 0.85,I_{Ex}\right),{\left(GP+AE\right)}_{x}\cdot 0.85\right]$$

**Figure 5:**
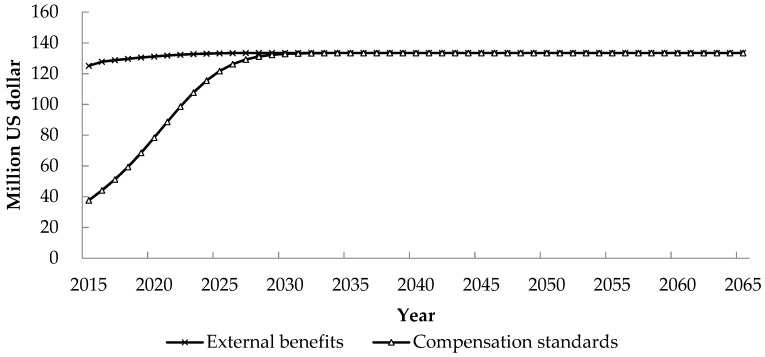
External benefits enjoyed and compensation standards provided by beneficiaries in E city.

The changing trends of the external benefits enjoyed by E city brought by the Z hydropower development and their compensation standards after enjoying the external benefits are shown in Figure 5.

The development of the Z hydropower project will bring the external benefits of grid performance and atmospheric environmental improvement for the power receiving areas. Due to the gradual increasing of the benefit in atmospheric environment improvement, the total benefits enjoyed by E city is increasing from 125 million US dollars in 2015 to 133 million US dollars in 2026, and then tends to be constant. After enjoying these external benefits, the beneficiaries in the power receiving area shall start to provide compensation after the normal operation of the hydropower project, and provide funds for the protection of environmental sensitive areas and the restoration of river ecosystems. As the effect of emission reduction becomes increasingly prominent, the country attaches more importance to the ecological environment and people’s living standards improvement, so the compensation standard is also gradually improving. The adjusted compensation standard increases gradually, from 38 million US dollars in 2015 to 133 million US dollars in 2033.

## 4. Discussion

Hydropower development will bring huge positive and negative externalities, such as the stimulation of regional economic development, the upgrading of shipping performance, the improvement of grid performance, the regulation of local climate, the improvement of atmospheric environment, the increasing of environmental capacity of water, and the prevention and mitigation of disasters; the invisible loss of immigrants, the declining of aquatic resources, etc. In practice, negative externalities of hydropower projects are often emphasized [23,24,25,26], and hydropower development enterprises are required to implement ecological and environmental protection measures, and to protect the interests of immigrants [27,28]. However, for a long time, insufficient attention has been paid to the positive externalities of hydropower development. The return on the investment of hydropower development mainly comes from internal benefits, such as power generation, water supply and irrigation, while the external benefits are all provided free of charge as social benefits. Thus, the external benefits and their internalization are studied to relieve the pressure on the hydropower development enterprises, provide more funds for the compensation of those adversely affected and for the restoration and protection of the natural ecological environment, and to realize the goals of benefit sharing and sustainable development of hydropower development [29].

The benefit of stimulating the development of regional economy by hydropower development is well studied both qualitatively [30] and quantitatively [31,32,33], but they only studied the driving effect of a project on the growth of economic aggregate, which is not the external effect of economic growth. By using the theory of Pigovian Tax [34], the external benefit of simulating the development of regional economy is measured by the actual return when using tax revenue as the investment. The external benefit in the regulation of local climate is not immediate but emerging gradually, and it will reach to a new balance, so the plant growth process of typical vegetation around the reservoir is chosen to represent the changing trend of this benefit [35]. The external benefit in improving the atmospheric environment refers to the emission reduction of CO_2_ and SO_2_ by replacing thermal energy with hydro energy. However, the emission of GHG from the reservoir has to be considered, as proven very large by many scholars [36,37,38]. The external benefit of disaster prevention and mitigation is a potential benefit, and it will only manifest when encountering a disaster which cannot be defended before the construction of the dam [39]. This external benefit shall only be considered in such times, so it is not used to calculate the compensation standard provided by the beneficiaries. 

The external benefits brought by the development of the Z hydropower project change in the development process, as contrasted with the constant values [40]. During the construction period, the external benefit mainly manifests as the stimulation of regional economy and varies with the project investment and the construction intensity. During the operation period, other external benefits beside the stimulation of regional economy gradually manifest, and the total external benefit is increasing gradually. The beneficiaries of the Z hydropower development include governments, enterprises and residents in the surrounding and downstream areas of the dam, as well as in the power receiving areas [41].

The beneficiaries in the surrounding and downstream areas enjoy the external benefits of regional economy stimulation, shipping performance upgrading, local climate regulation and water environmental capacity improvement. After enjoying these external benefits in 2006, the beneficiaries in the surrounding and downstream areas shall take out part of the external benefits they enjoyed and compensate those adversely affected by the project development. Considering people’s inadequate understanding of the benefit of these externalities and the limitation of their payment capacity and willingness, the compensation provided by these two areas increases slowly from 4 million US dollars in 2006 to 97 million US dollars in 2065. Although not all the external benefits are internalized, it can ease the tension on hydropower development enterprises and stimulate their enthusiasm in sharing their project benefit, and allow those adversely affected to enjoy the benefits of the project development.

The beneficiaries in the power receiving areas enjoy the external benefits of grid performance and atmospheric environment improvement. After enjoying these external benefits in 2015, the external beneficiaries in the power receiving area have the obligation to take out part of their external benefits to restore and protect the ecological environment in the project development area. Due to the high social development level of the power receiving areas, the adjustment coefficient change rapidly, reaching to the maximum of 1 in 2033, after which they are able to provide compensation in the amount of all the external benefits. Therefore, the adjusted compensation standard for the beneficiaries in the power receiving areas increases rapidly from 38 million US dollars in 2015 to 133 million US dollars in 2033. In the case of beneficiaries in the power receiving areas, all the external benefits are internalized after 2033.

The external beneficiaries in all three areas shall take out part of their external benefits, compensate those adversely affected by the project development, and restore and protect the ecological environment in the project development area. The compensation standards change with their awareness of the external benefits, their willingness to pay and ability to pay [42]. With the social and economic development, as well as the emphasis on the ecological environment, the beneficiaries’ willingness to pay and ability to pay increase gradually, so shall their compensation standards. The ways of sharing these external beneficiaries are not limited to one or two types. They can be realized in the form of increasing the tax rate for the beneficiaries in the surrounding and downstream areas, increasing the electricity prices for the beneficiaries in the power receiving areas, providing education, training, technical support, etc. The government shall urge and supervise the external beneficiaries in sharing their external benefits, and make policies and provide conveniences to facilitate the benefit sharing process.

Through defining, measuring and internalizing the external benefits, the social, economic, and ecological benefits brought by hydropower development can be properly redistributed. While hydropower development enterprises share their project benefits, external beneficiaries shall share their external benefits, too. This will lay a solid foundation for the coordinated and sustainable development of hydropower, and promote the construction of an ecological civilization and the harmonious development between human and nature. However, negative externalities and the effect of compensation are not discussed in this paper. In the future, the compensation effect shall be studied from the perspective of those adversely affected by the project development, and their marginal loss and marginal gain can be compared.

## 5. Conclusions

Hydropower development will bring vast internal and external benefits. To improve the traditional benefit-sharing mechanism of hydropower development enterprises sharing their project benefits, the external beneficiaries shall share their external benefits.

The governments, enterprises and residents in the surrounding areas and downstream areas of the dam enjoy the external benefits of regional economy stimulation, shipping performance upgrading, local climate regulation and water environmental capacity improvement since the beginning of the hydropower development. The external benefits vary with the project investment during the construction period, and gradually increase from 18 million US dollars in 2006 to 114 million US dollars in 2065. After enjoying these external benefits, they shall compensate those adversely affected by the project development, and the compensation standards increase from 4 million US dollars in 2006 to 97 million US dollars in 2065.

The governments, enterprises and residents in the power receiving areas enjoy the external benefits of grid performance and atmospheric environment improvement after the normal operation of the project. The external benefits they enjoyed increase from 125 million US dollars in 2015 to 133 million US dollars in 2026, and then tends to be constant. After enjoying these external benefits, they shall help to restore and protect the ecological environment in the project development area, and the compensation standards increase from 38 million US dollars in 2015 to 133 million US dollars in 2033, and then they are equivalent to the external benefits.

By applying an adjustment coefficient to the external benefits in constructing the dynamic compensation standard, a large sum of fund can be raised in each year without affecting the economic development of the beneficiaries, which can be used to provide more compensation for those adversely affected, and to provide more funds for the protection and restoration of the ecological environment. Benefit-sharing by both internal and external beneficiaries can promote the sustainable social and economic development, and the coordinated development between human and nature.

## Figures and Tables

**Table 1:** Engel’s coefficient of the all residential households in A county and D city.

Year	Engel’s Coefficient		Year	Engel’s Coefficient	
	A County	D City		A County	D City
2006	0.46	0.55	2010	0.45	0.44
2007	0.51	0.52	2011	0.45	0.49
2008	0.47	0.51	2012	0.43	0.51
2009	0.46	0.51	2013	0.42	0.47

**Table 2:** Engel’s coefficient of all residential households in E city.

Year	Engel’s Coefficient	Year	Engel’s Coefficient
2012	37.14	2015	27.29
2013	35.40	2016	25.53
2014	35.57	2017	25.15
